# Five-Years Review of *RHCE* Alleles Detected after Weak and/or Discrepant C Results in Southern France

**DOI:** 10.3390/genes13061058

**Published:** 2022-06-14

**Authors:** Pascal Pedini, Lugdivine Filosa, Nelly Bichel, Christophe Picard, Monique Silvy, Jacques Chiaroni, Caroline Izard, Laurine Laget, Stéphane Mazières

**Affiliations:** 1 Etablissement Français du Sang Provence-Alpes-Côte d’Azur-Corse, 13005 Marseille, France; pascal.pedini@efs.sante.fr (P.P.); lugdivine.deboisgrollier@efs.sante.fr (L.F.); nelly.bichel@efs.sante.fr (N.B.); christophe.picard@efs.sante.fr (C.P.); jacques.chiaroni@efs.sante.fr (J.C.); caroline.izard@efs.sante.fr (C.I.); laurine.laget@efs.sante.fr (L.L.); 2 Biologie des Groupes Sanguins, EFS, CNRS, ADES, Aix Marseille University, 13005 Marseille, France; silvy_monique@yahoo.fr

**Keywords:** *RHCE*, molecular biology, new variants, RHD*DAU-5, RHCE*ce *(1136)*, haplotype CE effect

## Abstract

Immunohematology laboratories are regularly facing transfusion issues due to serological weaknesses. Altered (partial) RH antigens account for most of them. In some situations, *RHCE* variant alleles are involved. Herein we present our three-step molecular exploration, with allele frequencies, that has efficiently untangled RH2 phenotype weaknesses and discrepancies in our 2017–2021 cohort. In the last 5 years, the PACA Corse EFS molecular platform received 265 samples from healthy blood donors or patients with C and C/e typing difficulties. The first-intention technique (DNA array and real time PCR for RHCE*CeRN research) detected *RHCE* variant alleles in 143 cases (54%). The *RHCE* alleles classically found in African populations were the most frequent, with RHCE*CeRN allele in 40 cases (15%) and *(C)ces* haplotype type 1 and 2 in 26 cases (10%). A “CE” effect haplotype was suspected in 56 cases, due to the uncommon DCE haplotype that may explain the low C expression. When there were no RHCE*Ce or RHCE*CE alleles, we then searched for *RHD* polymorphisms by DNA array. We detected the RHD*DAU5 and RHD*DIVa in 18 and 7 cases respectively, suggesting that C ambiguity is related to the presence of these alleles which has never been described with *DAU5*. If no variant *RHCE* and *RHD* alleles were detected, we finally sequenced the 10 exons of both *RHCE* and *RHD* genes according to the clinical context and found seven new *RHCE* alleles. Thus, this molecular strategy would improve the knowledge of *RHCE* variants’ expression and, thus, optimize the transfusion management.

## 1. Introduction

The RH blood group system is the most complex blood group system with more than 50 antigens encoded by the *RHD* and *RHCE* genes. These two genes, located on chromosome 1 at the *RH* locus, are a source of significant diversity, favored by their opposite orientation. Some variant Rh phenotypes are caused by exchange of genetic material between the two genes, resulting in many hybrid genes. Other phenotypes result from missense mutations. Over 200 *RHD* and 80 *RHCE* alleles have been reported (https://www.isbtweb.org/isbt-working-parties/rcibgt/blood-group-alleletables.html accessed on 20 May 2022). Variant alleles encode altered phenotypes with either reduced expression of antigen(s); lack of antigen(s); or expression of unexpected antigen(s) [1]. Immunohematology laboratories are regularly facing serologic weaknesses or discrepancies between two serologic techniques and only molecular biology can precisely characterize the molecular background explaining this feature.

In France, four molecular immunohematology laboratories (located in Brest, Creteil, Marseille, and Paris) from Etablissement Français du Sang (EFS) were implemented for specialized exploration of Rh phenotype inconsistencies. In Rh system, most serological issues involve RhD antigens and many studies have described the distribution of *RHD* variants in different populations [2,3,4,5,6]. The second most frequently concerned antigen is RhC antigen but few frequency studies are available apart from sickle cell disease patients and African cohorts [7,8,9].

The RhC weakening can be due to either RHCE*Ce or RHCE*CE variant alleles, the latter being less common. For RHCE*Ce variant allele the weakening usually affects both RhC and Rhe antigens. However, the common presence of a regular RHCE*ce in trans often covers the weakening of Rhe. As a result, simultaneous weakening of both antigens is only visible in the case of homozygous RHCE*Ce variants alleles or if a RHCE*cE allele is found in trans. Several RHCE*Ce variants were reported in either European or African populations [2,3,7,8]. These alleles are the result of SNPs (single nucleotide polymorphisms) in RHCE*Ce allele, or of hybrid *RHCE-D-CE* alleles. Some examples of RhC weak/discrepant results were found in samples lacking RHCE*C allele. In such cases, expression of RhC epitope(s) was linked to either SNP as in RHCE*01.36 and RHCE*01.28 [8] or to *RH* haplotypes as in *(C)ces type 1* and *type 2*, or *DIVa/ceTI* [10,11]. Lastly, RhC weakening is also reported in relation with the R_Z_ haplotype (RHD*01 associated with RHCE*04) referred to as “CE” effect [1].

Our objective in this study is to describe during a 5-year period (2017–2021) the diversity and distribution of variant *RHCE* encoding weak RhC or RhCe found in the molecular immunohematology laboratory of Marseille (southern France). The data will be useful to adapt the genotyping indications as well as the transfusion recommendations in case of serological weakening of the RhC and RhCe antigens.

## 2. Materials and Methods

### 2.1. Indications of Molecular Test for RHCE and Serology

Molecular analyses were performed in a molecular immunohematology laboratory based in EFS Marseille (France) using EDTA (ethylenediaminetetraacetic acid) blood samples from immunohematology laboratories in southern France and in Reunion Island (Indian Ocean). Samples were investigated because of RhC weakening or more rarely discrepant results between two phenotype techniques. A majority (95%) were from patients, the remaining being from donors.

First line and second line RhCE phenotype techniques were performed by using microplate with different clones (Table 1). The third line technique was the saline tube using clone MS273 for RhC, clone MS12 + MS260 for RhE, clone MS35 for Rhc, and clone MS62 + MS69 for Rhe (Eurobio, les Ulis, France). All methods were performed in accordance with the manufacturer’s recommendations.

### 2.2. Molecular Tests

Genomic DNA was extracted from 200 μL of whole blood using a Blood DNA mini kit (QIAamp, Qiagen, Courtaboeuf, France) according to the manufacturer’s instructions. BeadChip RhCE and RhD kits (BioArray Solution, Immucor, Warren, NJ, USA), through the detection of 28 and 36 polymorphisms respectively, allowed the identification of 48 and 67 alleles variants respectively. The analysis was performed in accordance with the manufacturer’s instructions.

The RHCE*02.10 (RHCE*Ce(RN)) allele was detected by allelic discrimination using TaqMan^®^ probes as previously described [12]. Polymerase chain reaction (PCR) amplification and sequencing of the *RHCE* and *RHD* genes coding regions were performed as previously reported [2]. Briefly, amplifications were performed on 100 ng of genomic DNA in a final volume of 50 μL containing PCR buffer, 2 mmol/L MgCl2, 80 ng/μL bovine serum albumin, 0.2 mmol/L of each dNTP, 0.05 units of Taq DNA polymerase (Invitrogen, Cergy Pontoise, France), and 200 nmol/L of each primer. Touchdown PCR was carried out in a thermocycler (Veriti, Applied Biosystems™, Foster City, CA, USA). After control of amplification on 1.5% (wt/vol) agarose gel, PCR products were sequenced by Eurofins Genomics (Paris, France). The reading of the sequences was done with the SeqmanPro software (DNASTAR).

### 2.3. Molecular Investigation Approach

**Figure 1:**
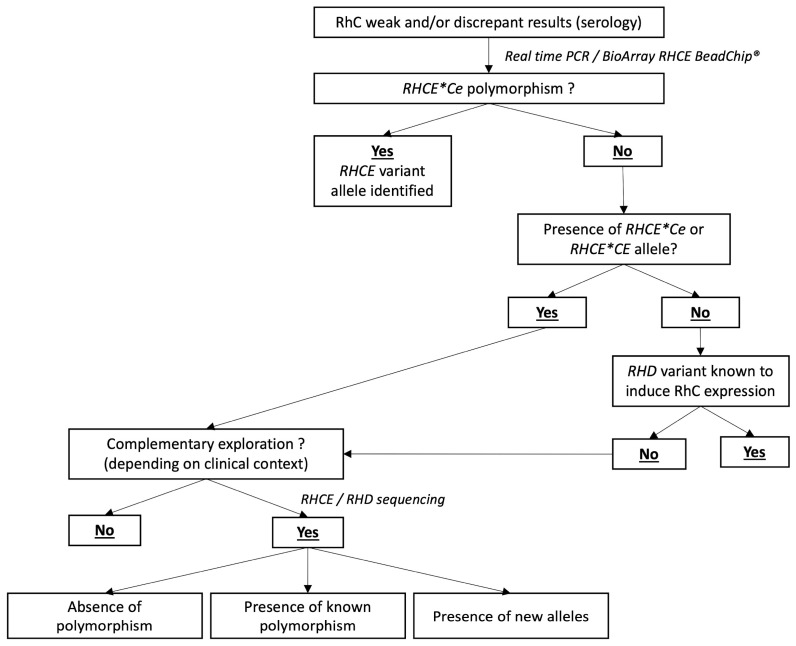
Workflow of molecular investigation.

The order of analyses is reported in Figure 1. Briefly, for all RhC (or RhCe) weakness and/or discrepant result, the first-line techniques consisted in using high throughput RHCE BeadChip DNA array. If no *RHCE* variant allele was identified in the presence of RH*Ce allele, allelic discrimination was performed to identify RHCE*02.10 variant.

If no variant was found by our first-line techniques, and if RHCE*C allele was detected and if the clinical context was relevant (young patients or polytransfused patients), the 10 exons of the *RHCE* gene were sequenced to identify polymorphisms not yet studied.

On the other hand, if no variant was identified with the first-line techniques and no RHCE*C was found, the RHD BeadChip was used to detect *RHD* variants which could explain the RhC serological result. If no such *RHD* allele was found, the 10 exons of the *RHD* gene were sequenced.

### 2.4. In Silico Analysis

Genetic sequences were aligned through the BLAST^®^ (NIH) database and submitted using the BankIt^®^ (NIH) application.

Mutation analysis and characterization of variants was performed with the Alamut Software Version 2.10.0 (Interactive Bio-software, Rouen, France). The Alamut software allowed us to name the variant according to the HGVS (Human Genome Variation Society) nomenclature (v15.11), to classify the variant according to the ACMG (American College of Medical Genetics) classification [13], and to query the dbSNP (v151) and gnomAD (v2.1) databases in order to know if the variant is yet described and to know its frequency. For exonic variant, the Alamut software has different calculation elements for the prediction of the pathogenicity of the variants: conservation of the nucleotide (phyloP), conservation of the amino acid, the physico-chemical difference between the amino acids (Grantham’s Dist.), and location of the amino acid variation in the protein. Finally, the Alamut software interrogates three prediction softwares, which are AlignGVGD (v2007), SIFT (v6.2.0), and MutationTaster (v2021). For intronic variations, the Alamut software allows splicing prediction by interrogating the MaxEnt, NNSPLICE, and HSF databases. Moreover, the mobidetails application (https://mobidetails.iurc.montp.inserm.fr/MD accessed on 13 April 2022) which queries the SPiP module (v2.1) [14] indicating a probability of alteration of the consensus splice site and Splice AI module [15] indicating a probability of losing the acceptor/donor sites was used.

In addition to the Alamut software, the prediction software UMD Predictor (v2022) [16] and Polyphen (v2) [17] were used.

## 3. Results

### 3.1. RHCE*Ce Alleles or Haplotype with a Known Impact on Rhc Serology

A total of 265 samples showed a weak RhC phenotype and/or discrepant results. In 38% (102/265), *RHCE* allele variants or haplotypes were identified using the first-intention techniques (Table 2). The most frequent *RHCE* alleles/haplotypes were RHCE*02.10 in 40 cases (15%), *(C)ces type 1* and *type 2* haplotypes in 9 and 17 cases, respectively, and the RHCE*02.01 (RHCE*Ce340T or RHCE*CeMA or RHCE*CeJAL) in 17 cases. Other variants were found in less than 3% of samples.

Nineteen *RHCE* alleles were identified by sequencing, as RHCE*02.18 in 11 cases (4%); RHCE*02.31 in 5 cases; RHCE*02.25 in 2 cases; and RHCE*02.16 and RHCE*02.11 in one case each.

All alleles were found to be heterozygous, except for the RHCE*02.10 which was homozygous in eight patients, thereby resulting in a rare RH: −46 RH:32 phenotype.

### 3.2. New RHCE Alleles

**Figure 2:**
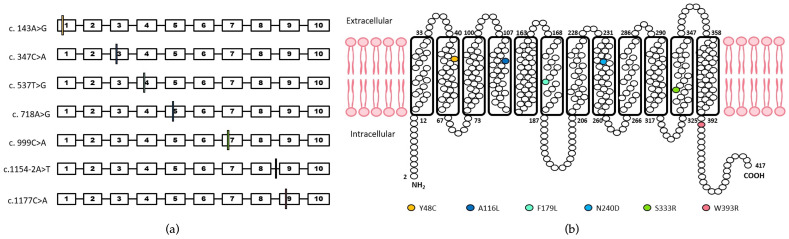
New variant RHCE*C alleles. (**a**) Position of polymorphisms on *RHCE* gene. Each box numbered from 1 to 10 represents one *RHCE* exon. The colored lines locate the different polymorphisms in the corresponding exons. The black line locates the intronic polymorphism in intron 8. (**b**) Representation of the RhCE protein in the red blood cell membrane and position of the changed amino acids. The 417 amino acids of the RhCE protein are represented by circles. Mature membrane proteins are missing the first amino acid. Amino acid substitutions identified in this report are color-coded. Model based on [18].

In eight samples, seven new RHCE*Ce alleles were identified (Figure 2, Table 3). The assignment of polymorphisms to RHCE*Ce allele was based on serology. All were in trans to conventional *RHCE*. Three of them were of Western European descent. The first allele was characterized by c.537T>G polymorphism encoding p.Phe179Leu, the second by c.718A>G polymorphism corresponding to p.Asn240Asp, and the third by c.999C>A corresponding to p.Ser333Arg. These variants were in the M5, M7, and M10 transmembrane segments, respectively. Different bio-informatics analysis predicted that the first substitution was probably damaging, and the two others were associated with low structural and functional impact.

One other polymorphism, c.143A>G (p.Tyr48Cys), was found in two unrelated individuals from the Reunion Island (Indian origin). The substitution was in the M1 transmembrane segment and the bioinformatics prediction scoring indicated a benign impact. Two others new alleles concerned two subjects from African ancestry, one in exon 9 with the c.1177T>C transition encoding p.Try393Arg with a benign bioinformatics prediction impact score, and the second showing a mutation in intron 8 c.1154-2a>t (IVS8-2a>t) which affects splice site.

Finally, one more patient, with unknown ethnic ancestry showed a c.347C>A substitution corresponding to p.Ala116Asp, in M3 transmembrane segment with a predicted benign impact according to bioinformatics scoring.

### 3.3. RHCE*ce Alleles Variants

In four cases lacking RHCE*C allele, RHCE*01.36 (ce307T) allele known to cause RhC weak and/or discrepant results by serology was found. More surprisingly, nine samples were heterozygous for an unusual allele, RHCE*ce1136T.

### 3.4. Haplotype CE Effect

In 62 patients (23%), no polymorphism was identified in first intentions techniques and sequencing and both RHCE*C and RHCE*E alleles were present. Twenty-five were typed RH-5 strongly suggesting that the weakening of RhC was the fact of the “CE” effect. This effect was suspected in the 37 remaining samples since we cannot determine whether the patient expresses Ce/cE or CE/ce haplotypes. Among them, 26 *RhCE* sequencing found no coding genetic defect.

### 3.5. RHD Alleles

In 59 cases (22%), *RHD* gene was investigated by BeadChip RhD because of the absence of RHCE*C allele found by first-line molecular biological technique. Eight-teen samples had RHD*10.05 allele (RHD*DAU5) and sequencing of *RHCE* revealed that nine of them also had RHCE*ce1136T allele (four were not sequenced for *RHCE*). Reactivity of these samples with anti-C antibodies is reported in (Table 4). Results showed weak and variable positive reactions of RBCs (Red blood cells) with some anti-C in samples bearing RHD*10.05 allele.

Six samples had RHD*04.01 allele (RHD*DIVa). The 35 remaining samples had no *RHD* variant identified by BeadChip RhD. Furthermore, among them, 12 RhD sequencing found no coding genetic defect.

## 4. Discussion

Over a five years period, our molecular immunohematology laboratory performed molecular exploration of 265 samples with weak and/or discrepant serologic results for RhC or RhCe. The serologic routine screening detects RhCE antigen weakening only when regular RhCE antigen is absent, making the prevalence of variants very hard to determine. In southwestern Germany, the cumulative frequency of *RHCE* alleles had been estimated between 0.2% and 0.012% using two approaches [8]. Another issue in determining real frequency was that the pattern of RhC reactivity varied from a negative reaction to high positive reaction (4+) in some cases, demonstrating that the reactivity against a precise RhCE antigen was variable according to both the clone and the method used.

Our study confirms that reduction in RhC reactivity is secondary to several molecular mechanisms. Indeed, it can be associated with low serologic reactivity due to the DCE haplotype (“CE” effect) in the absence of allelic *RHCE* variation, to RHCE*Ce variants, or to false positive serologic reactivity associated with RHCE*ce and/or *RHD* variants.

The most frequent *RHCE* variants in our study were those commonly found in African populations. Indeed, the RHCE*02.10 (RHCE*CeRN) allele was identified in 40 cases (15%) and *(C)ces type 1* and *type 2* in 26 cases (10%). They correspond to the main molecular backgrounds associated with partial RhC antigen in Sub-Saharan individuals though their frequencies vary by population [2,12,19]. As expected, the *(C)ces type 2* was more frequent than type 1 because it leads to a lower RhC reactivity. In fact *(C)ces type 1* sometimes does not result in weakening and typed as a regular RhC [11]. It should be noted that eight patients with the rare RH:-46 RH:32 phenotype were detected because of weak RhCe.

The other *RHCE* variants found in this study were described in populations of western European origin. The two most frequent variants, i.e., RHCE*02.01 (*CeMA*) (6%) and RHCE*02.18 (*Ce890C*) (4%), were found with higher frequencies compared to a previous French and two German studies [7,8,9]. Conversely, RHCE*02.04 (*CeVA*) had a higher frequency in the previous French study (4% versus <1% in our study) [9]. Thus, the distribution of variant *RHCE* encoding weak antigen reflects the multi-racial diversity of the southeast of France. This is highlighted by the different ethnicities of patients bearing the new *RHCE* alleles described herein.

As expected, the mechanisms underlying the variant *RHCE* alleles are like those observed for *RHD*. Indeed, they are caused by single or multiple nucleotide variations affecting the *RHCE* gene or by hybrid *RHCE-D-CE* alleles. These genetic alterations could induce conformational changes in the RhCE proteins as suggested by in silico analysis of the six novel alleles in our study. An impact on the intersubunit interactions within Rh complex at the membrane of RBC is a possibility that should also be considered. Alternative splicing alteration of the *RHCE* transcript or reduced translation as suspected for the intronic c.1154-2a>t mutation could also affect RhC antigen expression. However, the alloimmunization risk associated with these molecular defects could not be identified since none of these patients were exposed to regular RhCe polypeptides. Thus, while transfusion safety requires recognition of these variants in donors, no alloimmunization against the missing epitopes of recipients has been showed to date.

Approximately 20% of weak RhC antigen were suspected to be associated with the “CE” haplotype effect. Indeed, no molecular cause was demonstrated despite sequencing of the 10 exons and splice sites of *RHCE*. However, neither introns nor regulation regions were sequenced, and we cannot rule out the presence of a polymorphism affecting RhCE antigen expression. The uncommon DCE haplotype is found in individuals with the DCcEe or DCcEE phenotype. The number of available RhC antigen sites of CDE/cDE haplotypes was estimated as 8500–9800 per red cell, compared to 45,700–56,400 for the common CDe/Cde haplotypes [1,20]. In addition, the expression of RHCE*CE coding region in K562 cells showed normal expression of RhE antigen, but negative expression of RhC antigen, when tested with different anti-C MoAbs (monoclonal antibodies). This result suggests than the amino acid at position 226 (encoding E/e antigen) has a critical role in defining the epitope recognized by the anti-C MoAbs [21]. Thus, for the DCcEe phenotype, the use of anti-ce (RH6) or anti-CE (RH22) human antibodies or a transcriptional study will be necessary to determine the haplotypes. In any case, since the RhC antigen is not partial, there are no transfusion issue for the recipient. On the other hand, it is important to recognize them in donors.

One important finding of this study is that 59 patients with weak/discrepant RhC typing lack the RHCE*C allele and lack any RHCE*ce variant alleles known to be associated with RhC reactivity, i.e., RHCE*01.36 (RHCE*ce(P103S)) and RHCE*01.28 (RHCE*ce(X418Y)). The RHD*10.05 (RHD*DAU-5) allele associated or not with RHCE*ce1136T was found in 18 of these patients. These data support the fact that weak and variable positive reactions of RBCs with some anti-C are associated with the protein encoded by RHD*10.05. A further study with several anti-C clones should be carried out to specify the unexpected C reactivity. A similar variable and unstable reaction from nonreactive to 2+ was previously reported with many RHD*04.01/RHCE*01.02.01 samples (RHD*DIV/RHCE*ceTI) [10].

RHD*10.05 allele encodes a partial RhD phenotype with anti-D production [22]. A weak D reactivity was reported in routine typing of patients [23], however the 18 patients with RHD*10.05 typed regular D+ despite eight being homo- or hemizygous (RHD*01N.01/RHD*10.05). Similarly, despite an allele frequency of 1–2% in black donors, in African and in SCD (sickle cell disease) patients [19], this allele was not reported in studies of *RHD* variants in patients with weak/inconsistent RhD antigen.

These data provide another example of unexpected reactivity in RH blood group system. The practical impact of these unexpected reactions includes potential discrepancy between phenotype and DNA testing. It also suggests that C-ambiguity may be a warning signal for the detection of a partial RhD phenotype missed with anti-D.

Finally, 49 (18%) samples including 26 patients and 23 donors with weak RhC were molecularly unresolved. In this group, the *RhC* alleles without *RhE* alleles association were found in 14 samples. Among them, the *RHCE* sequencing was performed in seven cases without revealing molecular abnormalities, suggesting a weakening of RhC that may be secondary to the reagent used in automate because a single determination was performed or secondary to unknown transfusion recently performed interfering the RhCE detection. One other possibility is a possible genetic defect in regulatory regions of *RHCE* gene; *RHCE* transcriptional expression was not tested.

Thirty-five samples without *RHC* alleles were identified without molecular explanation. The *RHD* and *RHCE* sequencing did not showed molecular defect in 12 and 7 cases, respectively. As few *RHD* alleles, other molecular systems interacting with the Rh complex should produce false reaction with the reagent used.

## 5. Conclusions

In conclusion, these data highlighted the need to perform *RHCE* molecular analysis after weak and/or discrepant RhC serologic results. It should be noted that there are several dozen additional *RHD* and *RHCE* alleles that are considered to have no impact on the phenotype. In view of the high genotypic diversity of Rh system, it is necessary to distinguish between allelic variants that have an impact on the phenotype and those that are of clinical interest, i.e., a lack of epitope(s) associated to an alloimmunization risk. In the case of *RHCE* gene, the variants of clinical interest are for the most part rare “public negative” encountered in the population of Afro-Caribbean ancestry. This represents one of the major challenges in the transfusion management of sickle cell patients at the national and international level.

Transfusion management is therefore handled on an ad hoc basis, taking into account the prevention of alloimmunization (women of childbearing age), according to the patient’s phenotype, as well as other absent antigens that must be taken into account.

## Figures and Tables

**Table 1:** Clones used for first line and second line RhCE phenotype.

Technique		Clone			Supplier
	RhC	RhE	Rhc	Rhe	
Microplate	P3X25513G8 + MS24	906	MS33	P3GD512 + MS63	Qwalys, Diagast^®^, Loss, France
Gel column	MS24	MS260	MS33	MS16 + MS21 + MS63	IH500, Biorad, Hercules, CA, USA
	MS24	C2	MS42	MS16 + MS21 + MS63	AutoVue^®^ Innova Vision Max, Ortho Clinical Diag., Raritan, NJ, USA

**Table 2:** Alleles and haplotypes identified in 121 samples with weak/discrepant RhC serology using first intention techniques and RhCE sequencing.

Alleles	*n*
RHCE*02.10 (CeRN)	40
RHD*01N.06-RHCE*01.20.03 ((C)ces type 2)	17
RHCE*02.01 (CeMA ou CeJAL)	17
RHCE*02.18 (Ce890C)	11
RHD*03N.01-RHCE*01.20.03 ((C)ces type 1)	9
RHCE*02.03 (CeJAKH)	7
RHCE*02.22 (Ce667T)	5
RHCE*02.31 (Ce487-5G)	4
RHCE*01.36 (ce307T)	4
RHCE*02.25 (*Ce1007T*)	2
RHCE*02.04 (CeVA)	2
RHCE*02.16 (Ce728G)	1
RHCE*02.11 (Ce286A)	1
RHCE*02.08.01 (CeCW)	1

**Table 3:** New *RHCE* alleles.

*n*	Allele	Nucleotide and Amino Acid Changes	Phenotype	Ethnicity	RhC				Rhe				SNP ID *
					MS24	MS24	P3X25513G8 + MS24	MS273	MS16 + MS21 + MS63	MS16 + MS21 + MS63	P3GD512 + MS63	MS62 + MS69	
					Biorad	Ortho	Diagast	Eurobio	Biorad	Ortho	Diagast	Eurobio	
2	*RHCe(Y48C)*	c.143A>G p.Tyr48Cys	CcDee	Reunion Island		**+++**		**++**					rs758379880
1	*RHCe(A116N)*	c.347C>A p.Ala116Asp	CcDEe	unknown		**++**		**-**		**+++**		**-**	Bankit OM990677
1	*RHCe(F179L)*	c.537T>G p.Phe179Leu	CcDEe	Greece	**++**	**+**			**+**	**(+)**			Bankit OM990678
1	*RHCe(N240D)*	c.718A>G p.Asn240Asp	CcDEe	European	**+++**		**++++**		**++**		**-**		Bankit OM990674
1	*RHCe(S333R)*	c.999C>A p.Ser333Arg	CcDEe	European		**+++**	**?**			**++**	**?**		Bankit OM990679
1	*RHCe(IVS8-2A>T)*	c.1154-2A>T na	CcDee	Madagascar			**?**			**++**			Bankit 2562580
1	*RHCe(W393R)*	c.1177T>C p.Try393Arg	CcDEe	African		**++**							rs1374968969

*n*, number of observations; * identification in dbSNP database or submission number in Bankit; na, not applicable; -, negative result; (+), very weakly positive; ?, undetermined; +, weakly positive; ++, positive; +++, strongly positive; ++++ very strongly positive.

**Table 4:** *RHD* allele known or suspected of causing C-reactivity.

*RHD* Allele Identified		*n*
RHD*04.01 (DIVa)		6
RHD*10.05 (DAU5)	with RHCE*ce1136T	9
	without RHCE*ce1136T	5
	Non determined	4

## Data Availability

Not applicable.

## Abbreviations

EFS (Etablissement Français du Sang), SNPs (single nucleotide polymorphisms), EDTA (ethylenediaminetetraacetic acid), PCR (polymerase chain reaction), HGVS (Human Genome Variation Society), ACMG (American College of Medical Genetics), RBCs (red blood cells), MoAbs (monoclonal antibodies), SCD (sickle cell disease).

## References

[1] Daniels G. (2002). Human Blood Groups.

[2] Granier T., Beley S., Chiaroni J., Bailly P., Silvy M. (2013). A comprehensive survey of both RHD and RHCE allele frequencies in sub-Saharan Africa. Transfusion.

[3] Kappler-Gratias S., Auxerre C., Dubeaux I., Beolet M., Ripaux M., Le Pennec P.Y., Pham B.N. (2014). Systematic RH genotyping and variant identification in French donors of African origin. Blood Transfus..

[4] Zacarias J.M., Pereira E.M., Visentainer J.E., Guelsin G.A., de Melo F.C., Sell A.M. (2016). Frequency of RHD variants in Brazilian blood donors from Parana State, Southern Brazil. Transfus. Apher. Sci..

[5] Flegel W.A. (2007). Blood group genotyping in Germany. Transfusion.

[6] Flegel W.A., von Zabern I., Wagner F.F. (2009). Six years’ experience performing RHD genotyping to confirm D- red blood cell units in Germany for preventing anti-D immunizations. Transfusion.

[7] Bugert P., Scharberg E.A., Geisen C., von Zabern I., Flegel W.A. (2009). RhCE protein variants in Southwestern Germany detected by serologic routine testing. Transfusion.

[8] Döscher A., Vogt C., Bittner R., Gerdes I., Petershofen E.K., Wagner F.F. (2009). RHCE alleles detected after weak and/or discrepant results in automated Rh blood grouping of blood donors in Northern Germany. Transfusion.

[9] Pham B.N., Peyrard T., Juszczak G., Beolet M., Deram G., Martin-Blanc S., Dubeaux I., Roussel M., Kappler-Gratias S., Gien D. (2011). Analysis of RhCE variants among 806 individuals in France: Considerations for transfusion safety, with emphasis on patients with sickle cell disease. Transfusion.

[10] Westhoff C.M., Vege S., Halter Hipsky C., Hue-Roye K., Copeland T., Velliquette R.W., Horn T., Lomas-Francis C., Reid M.E. (2013). RHCE*ceTI encodes partial c and partial e and is often in cis to RHD*DIVa. Transfusion.

[11] Pham B.N., Peyrard T., Juszczak G., Dubeaux I., Gien D., Blancher A., Cartron J.P., Rouger P., Le Pennec P.Y. (2009). Heterogeneous molecular background of the weak C, VS+, hr B-, Hr B- phenotype in black persons. Transfusion.

[12] Tournamille C., Meunier-Costes N., Costes B., Martret J., Barrault A., Gauthier P., Galactéros F., Nzouékou R., Bierling P., Noizat-Pirenne F. (2010). Partial C antigen in sickle cell disease patients: Clinical relevance and prevention of alloimmunization. Transfusion.

[13] Richards S., Aziz N., Bale S., Bick D., Das S., Gastier-Foster J., Grody W.W., Hegde M., Lyon E., Spector E. (2015). Standards and guidelines for the interpretation of sequence variants: A joint consensus recommendation of the American College of Medical Genetics and Genomics and the Association for Molecular Pathology. Genet. Med..

[14] Leman R., Parfait B., Vidaud D., Girodon E., Pacot L., LE GAC G., Ka C., Ferec C., Fichou Y., Quesnelle C. (2022). SPiP: Splicing Prediction Pipeline, a machine learning tool for massive detection of exonic and intronic variant effect on mRNA splicing. Authorea.

[15] Bao S., Moakley D.F., Zhang C. (2019). The Splicing Code Goes Deep. Cell.

[16] Salgado D., Desvignes J.P., Rai G., Blanchard A., Miltgen M., Pinard A., Lévy N., Collod-Béroud G., Béroud C. (2016). UMD-Predictor: A High-Throughput Sequencing Compliant System for Pathogenicity Prediction of any Human cDNA Substitution. Hum. Mutat..

[17] Adzhubei I., Jordan D.M., Sunyaev S.R. (2013). Predicting functional effect of human missense mutations using PolyPhen-2. Curr. Protoc. Hum. Genet..

[18] Callebaut I., Dulin F., Bertrand O., Ripoche P., Mouro I., Colin Y., Mornon J.P., Cartron J.P. (2006). Hydrophobic cluster analysis and modeling of the human Rh protein three-dimensional structures. Transfus. Clin. Biol..

[19] Ba A., Beley S., Chiaroni J., Bailly P., Silvy M. (2015). RH diversity in Mali: Characterization of a new haplotype *RHD*DIVa/RHCE*ceTI(D2)*. Transfusion.

[20] Jia S., Chen J., Wen J., Wang Z., Wei L., Fu Y., Luo G., Ji Y. (2021). Serological screening and genetic analysis of *RhCE* variants in the Chinese Southern Han donors. Transfus. Med..

[21] Smythe J.S., Anstee D.J. (2001). Expression of C antigen in transduced K562 cells. Transfusion.

[22] Chen Q., Flegel W.A. (2005). Random survey for *RHD* alleles among D+ European persons. Transfusion.

[23] Souza Silva T.C., Cruz B.R., Costa S.S., Chiba A.K., Barros M.M.O., Langhi D.M., Bordin J.O. (2020). *RHD* and *RHCE* molecular analysis in weak D blood donors and in patients with Rh antibodies against their own corresponding Rh antigen. Blood Transfus..

